# mLST8 is essential for coronavirus replication and regulates its replication through the mTORC1 pathway

**DOI:** 10.1128/mbio.00899-23

**Published:** 2023-06-28

**Authors:** Yanan Fu, Zhen Fu, Zhelin Su, Lisha Li, Yilin Yang, Yubei Tan, Yixin Xiang, Yuejun Shi, Shengsong Xie, Limeng Sun, Guiqing Peng

**Affiliations:** 1 State Key Laboratory of Agricultural Microbiology, College of Veterinary Medicine, Huazhong Agricultural University, Wuhan, China; 2 Key Laboratory of Preventive Veterinary Medicine in Hubei Province, The Cooperative Innovation Center for Sustainable Pig Production, Wuhan, China; 3 Key Laboratory of Agricultural Animal Genetics, Breeding and Reproduction of Ministry of Education & Key Lab of Swine Genetics and Breeding of Ministry of Agriculture and Rural Affairs, Huazhong Agricultural University, Wuhan, China; 4 Key Laboratory of Prevention & Control for African Swine Fever and Other Major Pig Diseases, Ministry of Agriculture and Rural Affairs, Wuhan, China; 5 Hubei Hongshan Laboratory, Frontiers Science Center for Animal Breeding and Sustainable Production, Wuhan, China; Virginia Polytechnic Institute and State University, Blacksburg, Virginia, USA; University of Illinois Urbana-Champaign, Urbana, Illinois, USA

**Keywords:** mLST8, coronavirus, replication, mTORC1, autophagy

## Abstract

Coronaviruses (CoVs), which pose a serious threat to human and animal health worldwide, need to hijack host factors to complete their replicative cycles. However, the current study of host factors involved in CoV replication remains unknown. Here, we identified a novel host factor, mammalian lethal with sec-13 protein 8 (mLST8), which is a common subunit of mTOR complex 1 (mTORC1) and mTOR complex 2 (mTORC2), and is critical for CoV replication. Inhibitor and knockout (KO) experiments revealed that mTORC1, but not mTORC2, is essential for transmissible gastroenteritis virus replication. Furthermore, mLST8 KO reduced the phosphorylation of unc-51-like kinase 1 (ULK1), a factor downstream of the mTORC1 signaling pathway, and mechanistic studies revealed that decreased phosphorylation of the mTORC1 downstream factor ULK1 promoted the activation of autophagy, which is responsible for antiviral replication in mLST8 KO cells. Then, transmission electron microscopy indicated that both mLST8 KO and autophagy activator inhibited the formation of double-membrane vesicles in early viral replication. Finally, mLST8 KO and autophagy activator treatment could also inhibit the replication of other CoVs, indicating a conserved relationship between autophagy activation and CoV replication. In summary, our work reveals that mLST8 is a novel host regulator of CoV replication, which provides new insights into the mechanism of CoV replication and can facilitate the development of broad-spectrum antiviral drugs.

**IMPORTANCE:**

CoVs are highly variable, and existing CoV vaccines are still limited in their ability to address mutations in CoVs. Therefore, the need to improve our understanding of the interaction of CoVs with the host during viral replication and to find targets for drugs against CoVs is urgent. Here, we found that a novel host factor, mLST8, is critical for CoV infection. Further studies showed that mLST8 KO inhibited the mTORC1 signaling pathway, and we found that autophagy activation downstream of mTORC1 was the main cause of antiviral replication in mLST8 KO cells. Autophagy activation impaired the formation of DMVs and inhibited early viral replication. These findings deepen our understanding of the CoV replication process and provide insights into potential therapeutic applications.

## INTRODUCTION

Coronaviruses (CoVs) are enveloped positive-stranded RNA viruses grouped into four genera (α, β, γ, and δ) that primarily infect birds and mammals (1, 2). In the past few decades, CoV has developed into a pathogenic pathogen that seriously threatens human health. To date, seven human CoVs have been identified (3). Severe acute respiratory coronavirus 2 (SARS-CoV-2), which is currently of the greatest concern to humans, belongs to the Betacoronavirus genus and has led to a global pandemic (4). In addition, CoVs cause a series of animal infectious diseases, some of which cause economic losses in the livestock industry. For example, porcine transmissible gastroenteritis virus (TGEV), which belongs to the Alphacoronavirus genus, causes acute enteritis in swine of all ages and 100% mortality in piglets younger than 2 weeks old (5, 6). Despite extensive global research efforts to develop vaccines against CoVs, CoV variants that evade vaccine-induced immune neutralization are emerging and spreading rapidly. In this context, a comprehensive understanding of the interactions of CoVs with host factors is urgently needed to develop new broad-spectrum antiviral therapies.

Recent studies have identified some host factors and signaling pathways that are essential for the CoV life cycle (7 - 10). These include pathways related to cell proliferation and apoptosis, such as the ErbB and hypoxia-inducible factor 1 (HIF-1) pathways, and pathways related to innate immune responses, such as the tumor necrosis factor (TNF), nod-like receptor, and retinoic-acid-inducible gene I (RIG-I) signaling pathways (11, 12). Among the factors in these pathways, the HIF-1 signaling pathway factor HIF-1a can mediate hypoxia signaling to inhibit SARS-CoV-2 replication and virion production (13). The blockade of nuclear factor kappa-B (NF-kB) expression, the main signal transduction molecule in TNF signaling, can effectively inhibit the replication of CoVs (14). RIG-I is an inhibitor of SARS-CoV-2 infection in the early stages (15). These studies indicate that host factors are critical for CoV replication, but due to the complexity of the CoV proteome and replication cycle, the study of host factors involved in CoV replication is still at an early stage.

Mammalian lethal with sec-13 protein 8 (mLST8), a 34-kDa protein consisting of seven WD40 repeats, is a common subunit of mTORC1 and mTORC2 that is critical for the proper regulation of the mechanistic target of rapamycin (mTOR) signaling pathway (16 - 18). Based on the current literature, mLST8 has been studied more extensively in cancer than in viral biology (19, 20), and research on its role in viral biology is still preliminary. We previously found significant enrichment of the host factor mLST8 in a CRISPR screen (21), suggesting that mLST8 plays an important role during viral infection.

In the present work, we used TGEV as a model virus to study the role of the host factor mLST8 in CoV replication. We found that mLST8 knockout (KO) significantly inhibited CoV replication. Further investigation found that mLST8 KO inhibited viral replication in a manner dependent on mTORC1 signaling but not the mTORC2 signaling pathway. Notably, we found that mLST8 KO in PK-15 cells could inhibit viral replication, mainly due to the activation of autophagy downstream of mTORC1. Moreover, mLST8 KO and autophagy activation impaired the formation of double-membrane vesicles (DMVs) and inhibited early viral replication. These findings provide potential therapeutic targets for CoVs and deepen our understanding of the CoV replication mechanism.

## RESULTS

### mLST8 is a host factor required for TGEV replication

**Fig 1:**
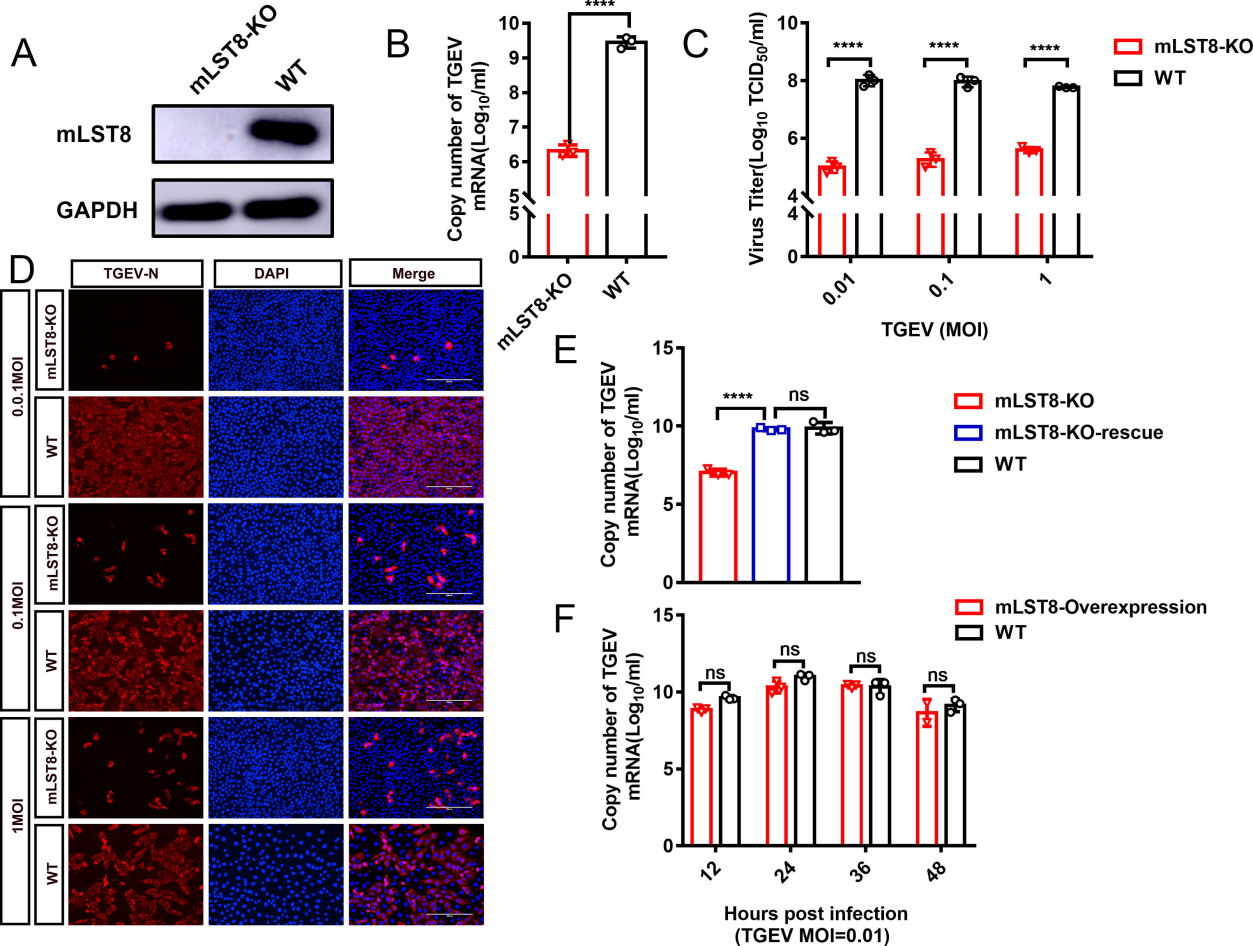
mLST8 is a host factor required for TGEV replication. (A) Western blot analysis validated the protein expression level of endogenous mLST8 in mLST8 KO cells and WT cells. GAPDH was used as an internal control gene. (B) mLST8 KO and WT cells were infected with TGEV at an MOI of 0.01. The TGEV N gene copy number was assessed by quantitative real-time PCR. (C and D) mLST8 KO and WT cells were infected with TGEV at different MOIs (1, 0.1, 0.01), and TGEV titers were tested at 24 hpi (hour post infection). Immunofluorescence assay was used to detect N protein expression in mLST8 KO cells following infection with TGEV at 24 hpi. Scale bar, 200 µm. (E) Rescue assays for WT, mLST8-KO, and mLST8-KO-rescue cells were infected with TGEV (MOI = 0.01). RT-qPCR was used to determine the expression of the TGEV N gene. (F) mLST8-overexpressing and WT cells were infected with TGEV (MOI = 0.01) for different durations (12, 24, 36, and 48 hpi). The TGEV N copy number was assessed by quantitative real-time PCR. The means and SDs of the results from three independent experiments are shown. ns, not significant, ***P  <  0.001; ****P  <  0.0001.

To explore whether mLST8 affects TGEV replication, we generated a clonal mLST8-KO PK-15 cell line through the CRISPR/Cas9 gene editing system. Sequence analysis showed that the mLST8 KO cell line contained a 1-nucleotide deletion (Fig. S1A), and western blot analysis showed that mLST8 was detected in the wild-type (WT) PK-15 cells but not in mLST8 KO cells, indicating successful construction of the mLST8 KO cell line (Fig. 1A). In addition, there was no difference in cell proliferation or viability between mLST8 KO and WT cells in the 5-ethynyl-2’-deoxyuridine (EdU) fluorescence assay, (3-(4, 5-dimethylthiazol-2-yl)-5-(3-carboxymethoxyphenyl)-2-(4-sulfophenyl)-2H tetrazolium) (MTS) assay, and Cell Counting Kit-8 assay (Fig. S1B through D). Subsequently, we used real-time quantitative PCR (RT-qPCR) to quantify the viral RNA from TGEV-infected WT and mLST8 KO cells (Fig. 1B). TGEV RNA replication was significantly decreased in the mLST8 KO cells after infection at an multiplicity of infection (MOI) of 0.01. Our results also showed that the viral titers of mLST8 KO cells were significantly reduced after TGEV infection at MOIs of 0.01, 0.1, and 1 (Fig. 1C). This finding was consistent with the results of immunofluorescence assays, which showed that the expression of the TGEV-encoded nucleocapsid (N) protein in the mLST8 KO cell line was reduced following TGEV infection at MOIs of 0.01, 0.1, and 1 (Fig. 1D). Further investigation of the TGEV viral titers in infected mLST8 KO cells at 12, 24, and 36 hours post-infection revealed that the viral titers of mLST8-KO cells were lower than those of WT cells at different time points (Fig. S1E).

To further detect whether mLST8 is required for TGEV infection, we constructed the mLST8-KO-rescue cell line using lentivirus-mediated transduction (Fig. S1F). As shown in Fig. 1E, TGEV replication in the mLST8-KO-rescue cells could be completely restored compared with that in the WT cells. Furthermore, to determine whether overexpression of mLST8 could modulate TGEV replication, an mLST8-overexpressing cell line was constructed (Fig. S1G). The overexpression of mLST8 in WT cells did not affect the replication of TGEV (Fig. 1F). These results indicated that mLST8 is a critical, basic host factor necessary for TGEV replication.

### mLST8 KO inhibits the early-stage replication of TGEV

**Fig 2:**
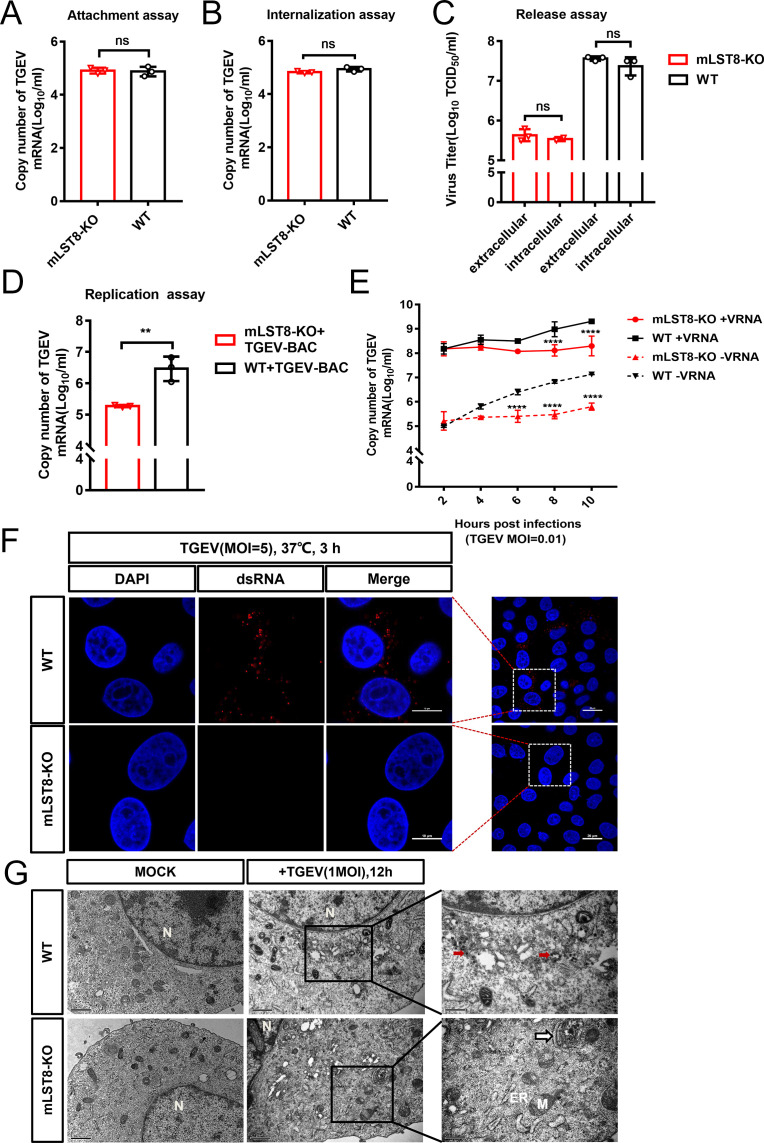
mLST8 KO inhibits the early-stage replication of TGEV. (A) mLST8 KO and WT cells were infected with TGEV (MOI = 5) at 4°C for 1 hour and assessed for TGEV adsorption. The cells were harvested and viral RNA was extracted to determine virion attachment at the cell surface. (B) mLST8 KO and WT cells were infected with TGEV (MOI = 5) at 4˚C for 1 hour, followed by incubation at 37°C and pronase treatment. The internalization activity of TGEV in mLST8 KO and WT cells was evaluated by absolute quantitative real-time PCR. (C) TGEV release in mLST8 KO and WT cells infected with TGEV (MOI = 0.01) were assessed. Intracellular and extracellular viral titers at 24 hpi were assessed by virus TCID_50_ assays. (D) mLST8 KO and WT cells were transfected with infectious TGEV-BAC plasmids. After 8 hours, they were incubated with the TGEV S protein antibody, and after 48 hours, the cells were collected and subjected to RT-qPCR. (E) mLST8 KO and WT cells were infected with TGEV at an MOI of 0.01. Positive (+vRNA) or negative (−vRNA) viral RNA was quantified by absolute quantitative real-time PCR. (F) Confocal microscopy analysis was used to evaluate the stages of TGEV replication by detecting dsRNA formation in WT and mLST8 KO cells at 3 hpi with TGEV (MOI = 5). Scale bars, 10 or 20 µm. (G) The effects of mLST8 KO cells on viral particle assembly were evaluated by TEM. Unlike WT cells, no virus-like particles (red arrows) were found in mLST8 KO cells. Scale bars, 2 µm or 500 nm as indicated. The means and SD of the results from three independent experiments are shown. ns, not significant; **P  <  0.01, ****P  <  0.0001.

To explore the role of mLST8 in the TGEV infection cycle, we first examined whether mLST8 affects the binding or entry of the virus. mLST8 KO and WT cells were incubated with TGEV for 1 hour at 4°C to ensure that the virus had been absorbed. The adsorption efficiency was detected by RT-PCR, which showed that the deletion of mLST8 did not affect the adsorption capacity of the viral particles (Fig. 2A). In the internalization assay, we used pronase and verified that pronase could remove most of the particles attached to the cells (Fig. S2). The internalization assay showed no difference in viral RNA levels between WT and mLST8 KO cells, implying that mLST8 does not play a role in viral internalization (Fig. 2B). We further examined the expression of the functional receptor porcine aminopeptidase N (pAPN) of TEGV by RT-PCR (22). The expression of pAPN did not differ between the mLST8 KO cells and WT cells (Fig. S3). The above results indicated that mLST8 KO did not affect the invasion stage of TGEV infection. Furthermore, the viral release assay showed that extracellular and intracellular viral titers of mLST8 KO cells were approximately equal and both were lower than those of WT cells (Fig. 2C), indicating that mLST8 KO did not inhibit the release of TGEV.

To determine whether mLST8 regulates TGEV genome replication, mLST8 KO and WT cells were transfected with TGEV-BAC plasmids to measure replication when entry was bypassed. Compared to that in WT cells, the RNA synthesis of TGEV in mLST8 KO cells was significantly decreased (Fig. 2D). Meanwhile, we used strand-specific RT-PCR to distinguish the production of positive-strand and negative-strand viral RNA (+vRNA and −vRNA, respectively). Intracellular +vRNA and −vRNA copies at 4 hpi (hour post infection) were increased in WT cells, while in mLST8 KO cells, the production of +vRNA and −vRNA was significantly inhibited (Fig. 2E). Confocal microscopy showed that the synthesis of double-stranded RNA (dsRNA), a marker of the TGEV RNA genome, was inhibited in mLST8 KO cells at 3 hours post-TGEV infection (Fig. 2F). We then evaluated the effect of mLST8 KO on viral particle assembly by transmission electron microscopy (TEM). The results showed that viral particle formation was hardly observed in mLST8 KO cells, as indicated (Fig. 2G), suggesting that mLST8 KO had severely impaired virion production. Taken together, these results suggested that mLST8 KO suppressed the early-stage replication of TGEV and further affected the formation of virions.

### mTORC1, but not mTORC2, is essential for TGEV replication

**Fig 3:**
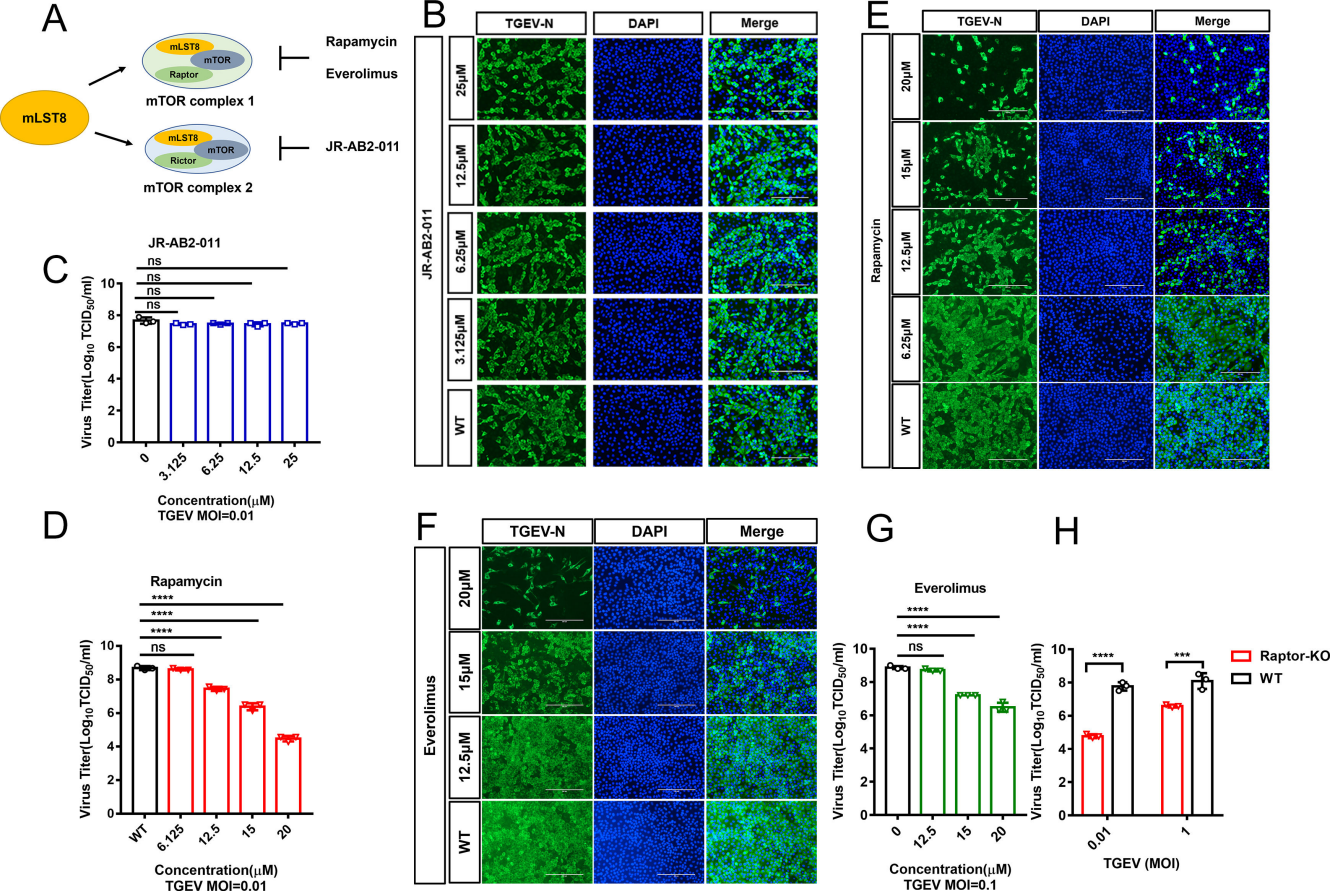
mTORC1, but not mTORC2, is essential for TGEV replication. (A) The diagram showed that mLST8 is a common subunit of mTORC1 and mTORC2. Rapamycin and everolimus are specific inhibitors of mTORC1, and JR-AB2-011 is a specific inhibitor of mTORC2. (B and C) PK-15 cells were incubated with different concentrations (25 µM, 12.5 µM, 6.25 µM, 3.125 µM) of JR-AB2-011 in advance for 2 hours, and cells treated with the inhibitor were infected with TGEV (MOI = 0.01). The TGEV titers were tested at 24 hpi (B). The TGEV N protein was detected by immunofluorescence assays. Scale bar, 200 µm (C). (D and E) PK-15 cells were incubated with different concentrations (20 µM, 15 µM, 6.25 µM) of rapamycin in advance for 2 hours, and cells treated with the inhibitor were infected with TGEV (MOI = 0.01). The TGEV titers were tested at 24 hpi (E). The TGEV N protein was detected by immunofluorescence assays. Scale bar, 200 µm (E). (F and G) PK-15 cells were incubated with different concentrations (20 µM, 15 µM, 12.5 µM) of everolimus in advance for 2 hours, and cells treated with the inhibitor were infected with TGEV (MOI = 0.1). The TGEV N protein was detected by immunofluorescence assays. Scale bar, 200 µm (F). The TGEV titers were tested at 24 hpi (G). (H) Raptor KO and WT cells were infected with TGEV at different MOIs (1 and 0.01), and the TGEV titers were tested at 24 hpi. The means and SDs of the results from three independent experiments are shown. ns, not significant; ***P  <  0.001; ****P  <  0.0001.

Among mTOR-interacting proteins, mLST8 is a conserved core component common to both the mTORC1 and mTORC2 complexes (16) (Fig. 3A). To determine whether mLST8 KO inhibits TGEV replication by regulating the mTOR signaling pathway, we examined the role of mTOR in viral replication. We initially monitored the role of mTORC2 in TGEV replication, and infected PK-15 cells were treated with JR-AB2-011, which inhibits mTORC2 activity by blocking the Rictor-mTOR association (23). Cell proliferation was not affected by the presence of JR-AB2-011 (Fig. S4A), and the replication capacity of TGEV was not significantly altered (Fig. 3B and C). These results indicated that TGEV replication is independent of mTORC2 activity.

Furthermore, we evaluated whether mTORC1 activity is required for TGEV replication. Infected PK-15 cells were treated with the mTORC1 inhibitor rapamycin, which inhibits FRAP/mTOR activity by forming a complex with the immunophilin FKBP12 (24). Neither cell viability nor cell proliferation was affected by the presence of rapamycin (Fig. S4B and C), but TGEV viral titers were significantly diminished (Fig. 3D). The expression levels of the TGEV N protein at 24 hpi decreased with increasing doses of rapamycin, as shown by immunofluorescence assay (Fig. 3E). Meanwhile, we used another inhibitor of mTORC1, everolimus, to further validate the effect of the mTORC1 pathway on TGEV (25). The CCK-8 assay results showed that 12.5, 15, and 20 µM everolimus had no effect on cell viability or proliferation (Fig. S4D). In the presence of everolimus, the replication capacity of TGEV was significantly decreased (Fig. 3F and G). The above results suggested that mTORC1 activity is required for TGEV replication. In addition, because Raptor, a unique scaffolding subunit of mTORC1, can bind substrates and modulate the phosphorylation of mTOR (26, 27). The Raptor-KO cell line was constructed. No difference in cell viability or cell proliferation between Raptor KO and WT cells was detected by EdU fluorescence assays and CCK-8 assays (Fig. S4E and F). As shown in Fig. 3H, the replication capacity of TGEV was significantly lower in Raptor KO cells than in WT cells. Altogether, these results demonstrated that mTORC1, but not mTORC2, is essential for TGEV replication.

### mLST8 KO activates autophagy to suppress viral replication by inhibiting the mTORC1 signaling pathway

**Fig 4:**
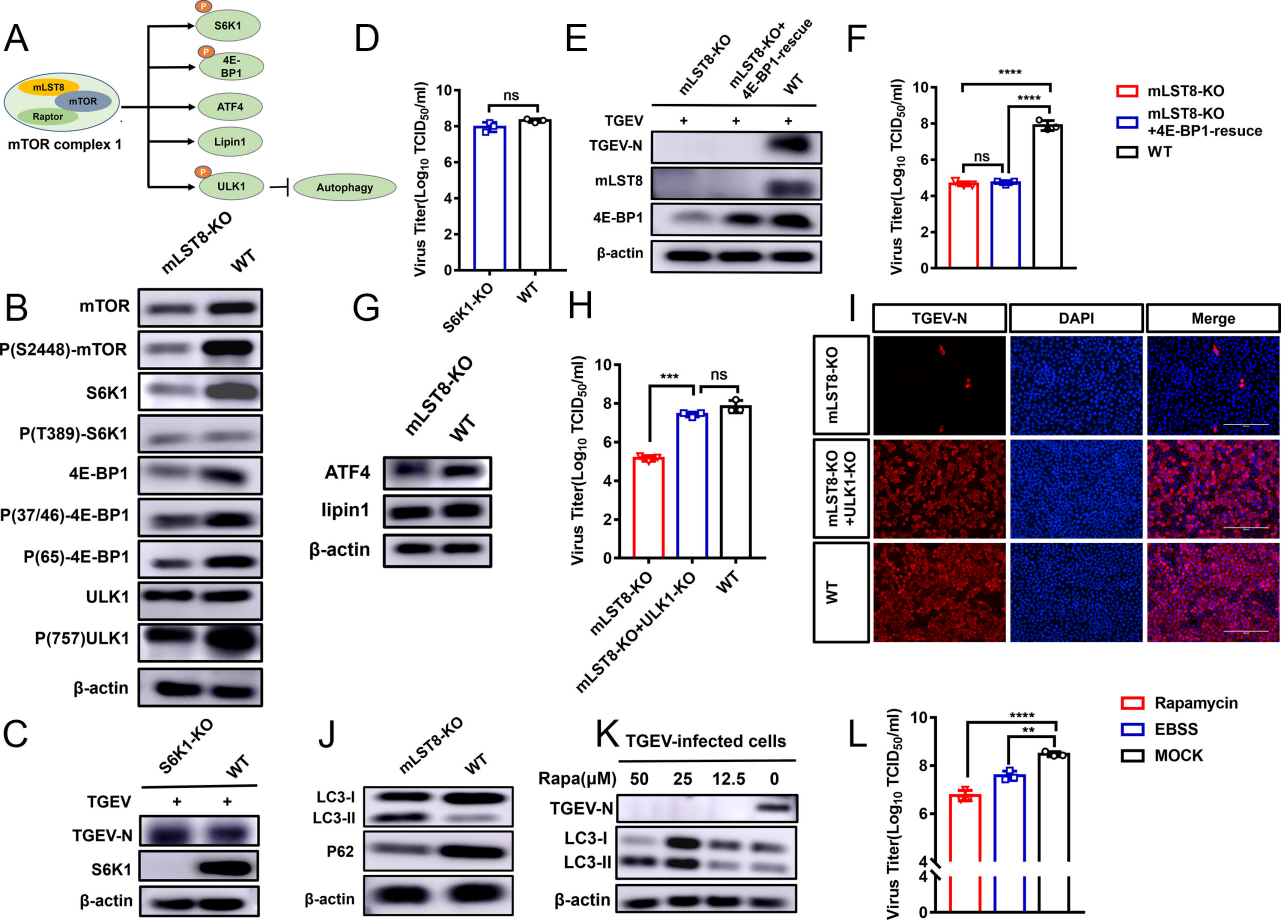
mLST8 KO inhibits the mTORC1 signaling pathway and enhances autophagy to inhibit viral replication. (A) The diagram shows the classic downstream substrate factors S6K1, 4E-BP1, lipin1, ATF4, and ULK1 in the mTORC1 signaling pathway. (B) The phosphorylation of mTOR, S6K1, 4E-BP1, and ULK1 and their protein expression in WT and mLST8-KO cells were detected by western blotting. (C) S6K1 KO and WT cells were infected with TGEV (MOI = 1), and the cells were lysed for western blotting at 24 hpi. (D) S6K1 KO and WT cells were infected with TGEV (MOI = 1), and the TGEV titers were tested at 24 hpi. (E) mLST8 KO, mLST8 KO + 4E-BP1-rescue, and WT cells were infected with TGEV (MOI = 0.01) at 24 hpi for detection. The cells were lysed for western blotting. (F) 4E-BP1 KO, mLST8 KO + 4E-BP1-rescue, and WT cells were infected with TGEV (MOI = 0.01), and the TGEV titers were tested at 24 hpi. (G) The expression of ATF4 and lipin1 in WT and mLST8-KO cells was detected by western blotting. (H) mLST8-KO, mLST8-KO + ULK1 KO, and WT cells were infected with TGEV (MOI = 0.01) for 24 hours. The TGEV titers were tested. (I) Immunofluorescence assays were carried out to detect N protein expression in mLST8 KO, mLST8 KO + ULK1 KO, and WT cells following infection with TGEV (MOI = 0.01) at 24 hpi. Scale bar, 200 µm. (J) Western blotting was used to detect the protein expression of LC3-II and p62 in WT and mLST8-KO cells. (K) Western blotting was used to detect the protein expression of LC3-II and the TGEV N protein in rapamycin-treated WT cells infected with TGEV (MOI = 0.01) at 24 hpi. (L) PK-15 cells were preincubated with rapamycin and earle's balanced salt solution (EBSS) for 2 hours, and the treated cells were infected with TGEV (MOI = 0.01). The TGEV titers were tested at 24 hpi. The means and SDs of the results from three independent experiments are shown. ns, not significant; ***P  <  0.001; ****P  <  0.0001.

mTORC1 regulates various biological processes through the phosphorylation of several proteins, such as substrate S6 kinase 1 (S6K1), eukaryotic translation initiation factor 4E (eIF4E)-binding protein 1 (4E-BP1), lipin1, transcription factor 4 (ATF4), and unc-51-like kinase 1 (ULK1) (Fig. 4A) (28, 29). Studies have found that mLST8 is a core subunit of the mTORC1 complex (16), but it was unclear whether mLST8 deletion would affect mTORC1 activity in PK-15 cells. In our study, when mLST8 was deleted, the protein expression level of S6K1 was greatly reduced, the phosphorylation of S6K1 (T389) was slightly reduced, and the phosphorylation and expression of 4E-BP1 (Thr37/Thr46/Ser65) were reduced. At the same time, phosphorylation of ULK1 (S757) was also reduced in mLST8 KO cells (Fig. 4B). The results suggested that mLST8 KO could inhibit the mTORC1 signaling pathway.

mTORC1 downstream substrates play a role in the regulation of translation, lipid biosynthesis, nucleotide biosynthesis, and autophagy. However, the exact mechanism by which mTORC1 regulates viral replication remains unclear. To clarify the underlying mechanisms by which mLST8 regulates viral replication, we first analyzed the roles of the key factor S6K1 downstream of mTORC1 in viral replication. S6K1 KO cells were constructed. Then, S6K1 KO and WT cells were inoculated with TGEV (MOI = 0.01). There was no significant difference in TEGV replication between the S6K1 KO and WT cells (Fig. 4C and D), suggesting that S6K1 is not essential for TGEV replication. Subsequently, we investigated whether the reduction in viral replication caused by mLST8 KO was regulated by 4E-BP1. 4E-BP1 expression was replenished in mLST8 KO cells (Fig. 4E). However, TGEV replication was still inhibited in the 4E-BP1-rescued mLST8 KO cells (Fig. 4E and F). These results suggested that mLST8 KO did not inhibit TGEV replication through S6K1 and 4E-BP1.

In parallel, mTOR can also regulate the synthesis of lipids and nucleotides. Previous studies have reported that mTORC1 can regulate lipid synthesis through lipin1 (30, 31). Western blot analysis showed no difference in lipin1 expression when mLTS8 was knocked out (Fig. 4G). We further detected cholesterol and lipid droplets (LDs) to explore the lipid synthesis in mLST8 KO cells and WT cells. The results showed no difference in the synthesis of cholesterol and LDs between mLST8 KO and WT cells (Fig. S5A and B), indicating that deletion of mLST8 in PK cells does not affect lipid synthesis. Activation of the mTORC1 signaling pathway can cause the ATF4 to accumulate, which enhances the production of methylenetetrahydrofolate dehydrogenase 2 (MTHFD2), leading to the increased production of nucleotides (2, 32). To investigate the effect of mLST8 KO on nucleotide synthesis, we examined the expression of ATF4 and MTHFD2 by western blot analysis and RT-qPCR, respectively. Although the expression of ATF4 was slightly decreased, the expression of the effector MTHFD2 was unchanged in mLST8 KO cells (Fig. 4G; Fig. S5C). These results suggested that mLST8 KO may not inhibit TGEV replication by regulating lipid or nucleotide synthesis.

Next, we turned our attention to ULK1, another factor downstream of mTORC1. We constructed mLST8 and ULK1 double KO PK-15 cells. Compared with those in mLST8 KO cells, the viral titers of TGEV were increased in mLST8 and ULK1 double-KO cells and were similar to those in WT cells. The expression of TGEV N protein was restored in mLST8 and ULK1 double-KO cells and was equivalent to the level in the WT cells (Fig. 4H and I). These results suggested that mLST8 KO inhibited TGEV replication through ULK1. ULK1 is an autophagy-initiating factor. Inhibition of mTORC1 leads to dephosphorylation of ULK1, which promotes the formation of the ULK1-ATG13-FIP200 complex and further activates autophagy (29, 33, 34). In our study, the phosphorylation of ULK1(S757) was largely reduced in mLST8 KO cells, suggesting that mLST8 KO may have activated autophagy. Subsequently, western blot results showed that the conversion of microtubule-associated protein 1 light chain 3 (LC3-I) to microtubule-associated protein 1A/1B-light chain 3 (LC3)-II (LC3-II) was significantly enhanced in mLST8 KO cells compared to WT cells. Consistently, sequestosome 1 (p62) levels in mLST8 KO cells were significantly lower than those in WT cells (Fig. 4J). The conversion of LC3-I to LC3-II, and degradation of p62 are indicators of autophagy (35). These results confirmed the enhanced ability of mLST8 KO to initiate autophagy. Following the activation of autophagy, the expression of TGEV N was inhibited, and the viral titers were decreased. At the same time, viral replication was also inhibited after starvation treatment with EBSS (earle's balanced salt solution) (Fig. 4K and L). In summary, these findings indicated that the knockout of mLST8 led to the inhibition of the mTORC1 signaling pathway, which in turn activated autophagy downstream of mTORC1 to inhibit viral replication.

### Activation of autophagy inhibits the formation of DMVs

**Fig 5:**
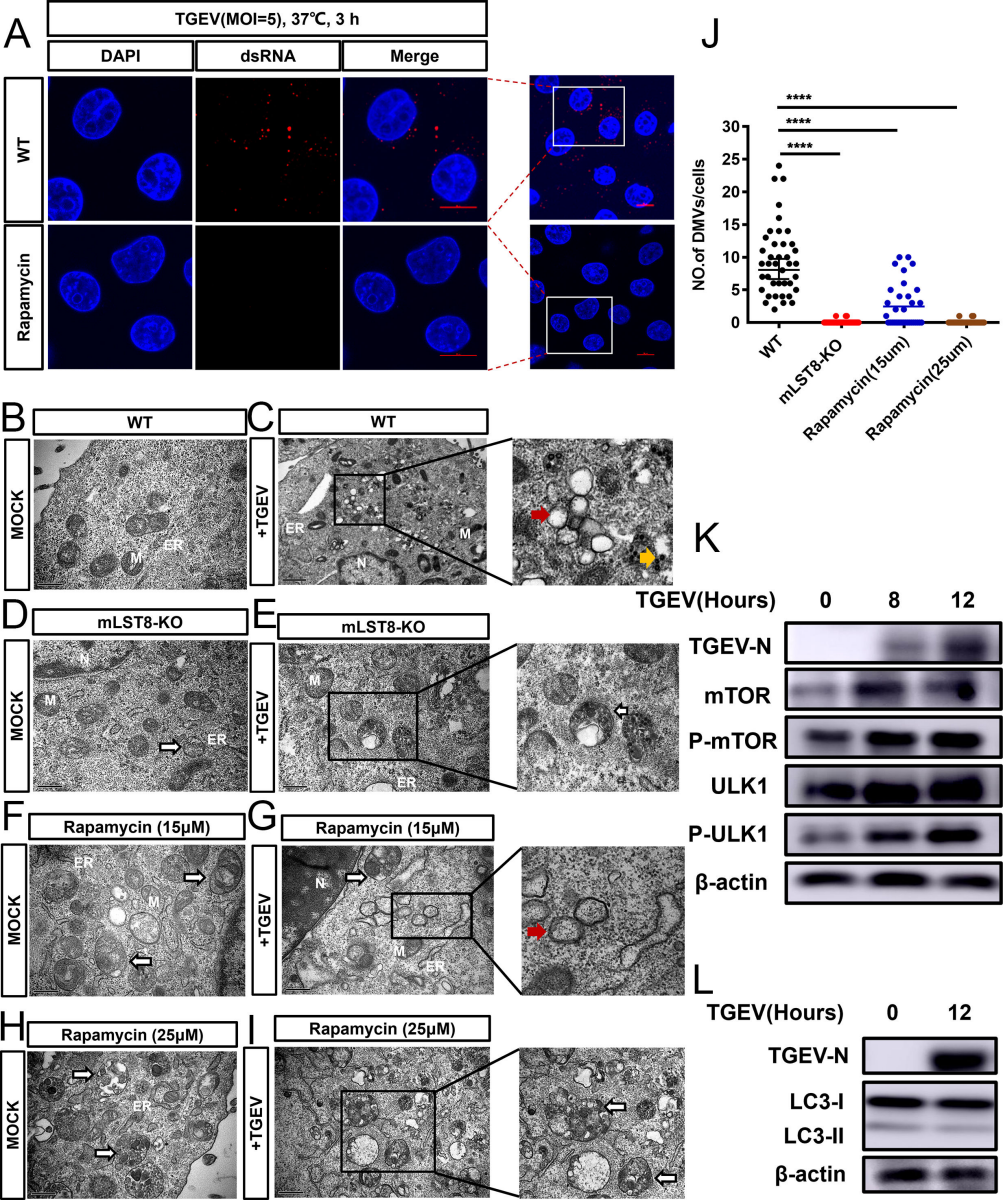
Activation of autophagy inhibits DMV formation. (A) Confocal microscopy analysis of dsRNA formation was used to assess the TGEV replication stage after rapamycin-treated and WT cells were infected with TGEV (MOI = 5) at 3 hpi. Scale bar, 10 or 20 µm. (B and C) The typical reduction in DMVs (diameter 150–300 nm) was observed by transmission electron microscope in TGEV-infected or uninfected PK-15 cells (MOI = 1, 12 hours) (red arrows). Scale bar, 500 nm. (D and E) Autophagosome-like vesicles (white arrows) were observed by electron microscopy in mock-infected and TGEV-infected mLST8 KO cells (MOI = 1, 12 hours). Scale bar, 500 nm. (F and G) PK-15 cells pretreated with 15 µM rapamycin were infected with TGEV at an MOI of 1 for 12 hours or left uninfected, fixed, and processed for electron microscopy analysis. (H and I) PK-15 cells pretreated with 25 µM rapamycin were infected with TGEV at an MOI of 1 for 12 hours or left uninfected, fixed, and processed for electron microscopy analysis. (J) Quantification of DMV numbers per cell is shown as the mean ± SD (*n* = 40). (K) PK-15 cells were infected with TGEV at an MOI of 1, and the cells were harvested at 8 hpi and 12 hpi. Representative western blots with the indicated antibodies are shown. (L) PK-15 cells were infected with TGEV at an MOI of 1, and cells were harvested at 12 hpi. Western blotting was used to detect the protein expression of LC3-II in TGEV-infected and uninfected cells. Two-tailed unpaired Student’s *t*-test, ****P < 0.0001. Red arrows indicate DMVs in the image, white arrows indicate autophagosome-like vesicles, and yellow arrows indicate viral particles in the image.

To investigate the mechanism by which autophagy activation inhibits CoV replication, we chose rapamycin as an autophagy activator, and autophagy was significantly activated in the presence of rapamycin. We found that the synthesis of viral dsRNA was significantly reduced in autophagy-activated cells (Fig. 5A). DsRNA mainly accumulates in DMVs, and CoV infection induces the formation of DMVs, which are the sites of viral RNA replication (36, 37).

To further investigate whether the activation of autophagy leads to the impaired formation of DMVs, we performed TEM to evaluate the formation of CoV replication organelles on TGEV infection. Unlike the results in uninfected controls (Fig. 5B), the formation of typical DMVs (150–300 nm in diameter) was found in TGEV-infected cells (Fig. 5C and J). Most of the DMVs contained a few fibrous materials in the inner vesicles. In contrast, typical DMV formation was hardly observed in mLST8 KO cells (Fig. 5E and J). We also observed the formation of autophagy-like vesicles (double- and single-membrane vesicles containing cytoplasmic components or sequestered organelles) in both mLST8 KO cells and TGEV-infected mLST8 KO cells, further suggesting that mLST8 KO led to the activation of autophagy (Fig. 5D and E). To examine the effect of autophagy activation on the formation of DMVs, we pretreated cells with different concentrations of autophagy activator for 2 hours and subsequently infected the cells with TGEV. TEM images showed that the number of autophagosome-like vesicles in the cytoplasm of cells treated with a high-dose autophagy activator was significantly greater than that of cells treated with a low-dose autophagy activator (Fig. 5F and H). Importantly, we found that small numbers of DMVs were formed after infection with TGEV of cells treated with low concentrations of autophagy activators (Fig. 5G and J). The formation of DMVs was not observed after infection with TGEV in the presence of a high concentration of autophagy activator (Fig. 5I and J). The above results suggested that the activation of autophagy inhibits the formation of DMVs. To further investigate the mTOR and autophagy status during TGEV infection, we infected PK-15 cells with TGEV and monitored the phosphorylation of mTOR and its downstream factor ULK1 at 8 and 12 hours post-infection. The results revealed a significant increase in the phosphorylation of both mTOR and ULK1 at 8 and 12 hours after TGEV infection (Fig. 5K). We further investigated the status of autophagy during TGEV infection by monitoring the conversion of the autophagy marker protein LC3-I to LC3-II. The results showed that autophagy was in an inactive status at 12 hours after TGEV infection (Fig. 5L). These results predicted that TGEV may inhibit the activation of autophagy to enhance its own replication at or before 12 hours of infection.

### mLST8 is a host factor for the replication of multiple coronaviruses

**Fig 6:**
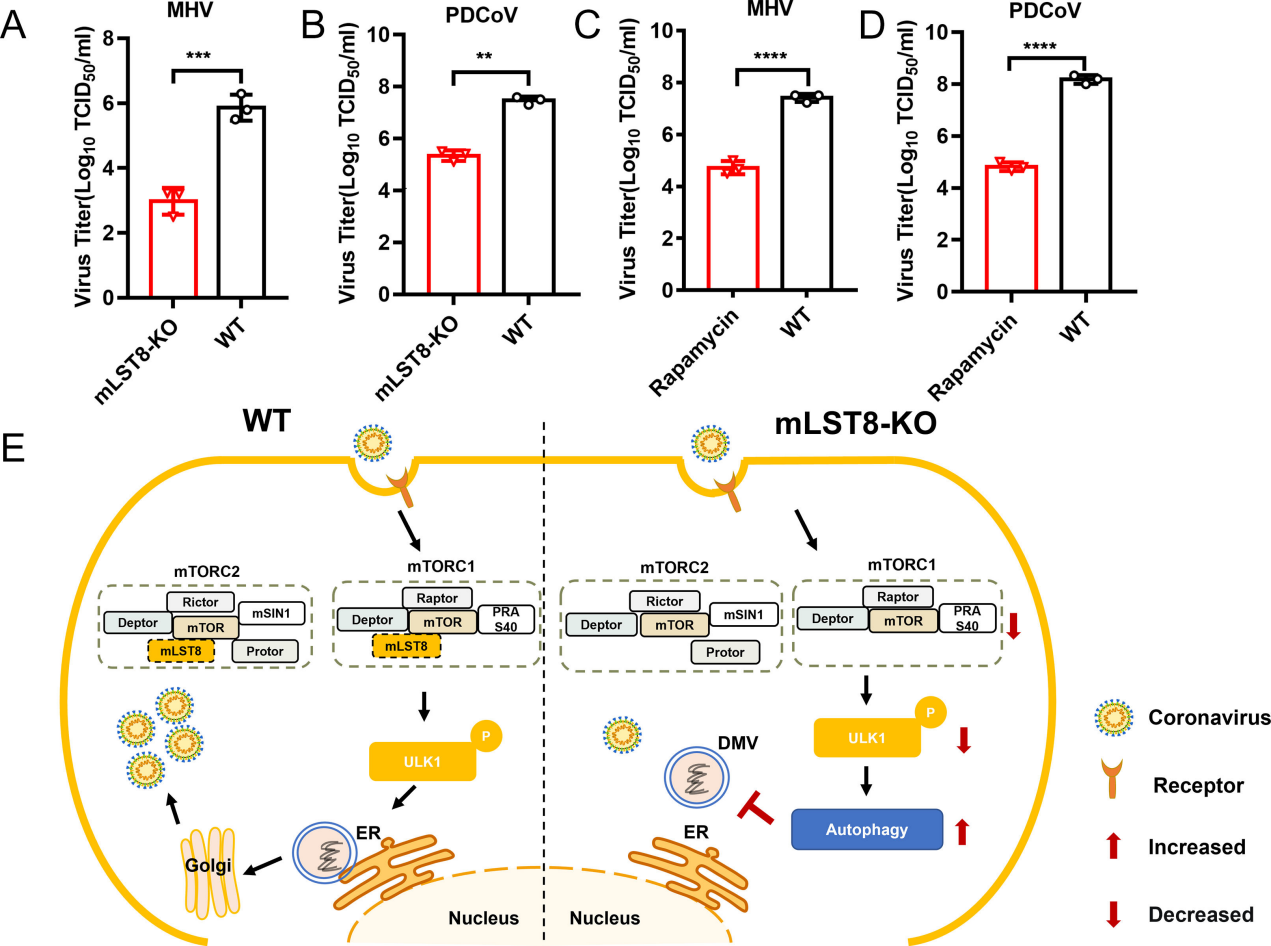
mLST8 is a host factor for the replication of multiple viruses. (A) mLST8 KO L929 and WT cells were infected with MHV (MOI = 0.01, 24 hpi), and the MHV titers were tested at 24 hpi. (B) mLST8 KO and WT cells were infected with PDCoV (MOI = 0.01, 24 hpi), and the PDCoV titers were tested at 24 hpi. (C and B) WT cells were preincubated with rapamycin at a concentration of 25 µM for 2 hours, and the cells treated with rapamycin were infected with MHV (MOI = 0.01) (C) or PDCoV (MOI = 0.01) (D). (E) A model illustrating the roles of mLST8 in the CoV replication cycle is shown. In WT cells, CoVs may utilize the mTORC1 pathway and promote their own replication. In mLST8 KO cells, the phosphorylation of ULK1, a downstream factor of the mTORC1 pathway, was reduced, leading to the activation of autophagy. Autophagy activation impaired DMV synthesis to defend against viral replication. **P  <  0.01; ***P  <  0.001; ****P  <  0.0001.

To verify the effect of mLST8 KO against other CoVs, we investigated the potential impact of mLST8 on Betacoronavirus mouse hepatitis virus A-59 (MHV-A59). Compared with those of WT L929 cells, the viral titers of MHV-A59 in mLST8 KO L929 cells were significantly decreased (Fig. 6A). The loss of mLST8 did not have an impact on cell viability or proliferation in L929 cells (Fig. S6A through C). We also found that porcine Deltacoronavirus (PDCoV) replication was significantly inhibited in mLST8 KO cells compared with WT cells (Fig. 6B). To further verify that mLST8 regulates viral replication by autophagy, WT cells were pretreated with rapamycin and subsequently infected with MHV-A59 and PDCoV. The results showed that autophagy activation inhibited the replication of MHV-A59 and PDCoV (Fig. 6C and D). These results revealed that mLST8 is an essential host factor for the replication of multiple CoVs. In summary, during replication, CoVs may utilize the mTORC1 signaling pathway to complete replication. However, in mLST8 KO cells, mTORC1 signaling was inhibited, which led to the decreased phosphorylation of downstream ULK1 and activation of autophagy. Enhanced autophagy then disrupted DMV formation to defend against viral replication (Fig. 6E).

## DISCUSSION

CoVs pose a serious threat to human health and economic development. Due to the continuous mutation of CoVs, vaccines have certain limitations in preventing CoV infection (38, 39). Further research on CoV interactions with host factors is urgently needed to identify new drug targets for CoV treatment. Based on a previous CRISPR screen carried out in our laboratory (21), we identified mLST8 as a novel host protein that is essential for CoV replication and systematically studied the possible conserved mechanism of mLST8 in CoV replication.

We found that mLST8 regulates CoV replication through the mTORC1 pathway. However, the exact function of mLST8 in the mTORC1 pathway is unclear (19, 40). In this study, we found that mLST8 KO inhibited mTORC1 signaling in PK-15 cells, resulting in decreased phosphorylation of the downstream factors 4E-BP1, S6K1, and ULK1. It is possible that mLST8 functions as a scaffold, and knockout of mLST8 may lead to changes in the catalytic activity and stability of the mTORC1, resulting in impaired substrate activation (16). Studies have found that the mTORC1 signaling pathway plays an important role in the replication of SARS-CoV-2 and Middle East respiratory syndrome coronavirus (MERS-CoV) (41 - 43). Similarly, we also found that mTORC1 signaling is important for other CoVs, such as TGEV, MHV-A59, and PDCoV. These results indicated that mTORC1 plays a conserved role in CoV replication. The mTORC1 signaling pathway is known to regulate host translation, autophagy, nucleotide synthesis, and lipid synthesis (42), On this basis, we identified autophagy as a key regulator of viral replication.

Autophagy is a conserved lysosome-based degradative pathway that is involved in many different aspects of cell biology, such as signal regulation (44 - 46). The role of autophagy in CoV replication is complex. On the one hand, CoVs utilize certain autophagy factors such as TMEM41B, class III PI3K complexes, and DFCP1 to participate in their replication (10, 21, 47, 48). However, knockout of the autophagy initiator ATG5 did not affect the replication of MHV, TGEV, and SARS-CoV-2 (21, 49, 50). In our study, the activation of autophagy inhibited TGEV replication, which is consistent with the effects of autophagy activation in other CoV studies (51, 52). In addition, our study further revealed that activation of autophagy can lead to impaired formation of DMVs. It has been reported that non-lipidated LC3-I is required for CoV-induced DMV formation and is associated with the outer membrane of DMVs, but LC3-II is not essential for the formation of DMVs (53). However, autophagy activation leads to the conversion of non-lipidated LC3-I to LC3-II, which may lead to a decrease in the autophagic factor LC3-I and further impair DMV formation. The structure of DMV is similar to that of autophagosomes and its formation depends on endoplasmic reticulum–derived membranes, as does autophagy (37, 54). Furthermore, the formation of DMV requires the involvement of some autophagy factors (48, 55, 56). Autophagy activation allows for the recruitment of autophagic factors into the autophagic pathway, which may reduce the recruitment of these factors by the virus and thus lead to impaired formation of DMV. In addition, DMV formation requires the participation of viral proteins and associated host proteins (57, 58). However, the activation of autophagy can degrade the viral proteins and host proteins that are involved in viral replication (59 - 61). Therefore, it is also possible that the activation of autophagy degrades viral proteins and host proteins required for the formation of DMVs.

Our findings revealed that TGEV activated mTORC1 at 12 hours of infection or earlier, and further inhibited the activation of autophagy, and the replication of SARS-CoV-2 and MERS-CoV also inhibited the maturation of autophagy (41, 52, 62). Therefore, we proposed that coronaviruses may activate the mTORC1 pathway and further inhibit autophagy activation, which facilitates their own replication. However, the exact mechanism remains to be investigated.

In summary, our work has identified a novel host factor, mLST8, that regulates TGEV replication. Further, we found that mLST8 KO activated autophagy downstream of mTORC1 to impair DMV synthesis to defend against viral replication. Our findings indicated that mLST8 may be a host-directed target to inhibit the replication of emerging and re-emerging CoVs.

## MATERIALS AND METHODS

### Plasmid construction

The lenti-sgRNA-EGFP vector, a lentiviral sgRNA expression vector, was digested by the *Bbs*I restriction enzyme (NEB). The lentiCRISPR-V2 vector was digested by the *BsmB*I restriction enzyme (NEB). Individual sgRNAs were cloned into the lenti-sgRNA-EGFP vector and lentiCRISPR-V2 vector for validation in PK-15 cells and L929 cells, respectively. For rescue and overexpression experiments, to reduce the role of sgRNA and Cas9 in mLST8-KO cells, mLST8 was mutated at a specific base in the protospacer adjacent motif sequence, which did not alter the encoded amino acid. A point mutation was induced in the sequence of mLST8, and the mutant sequence was cloned into the pLVX-T2A-mCherry-Puro vector (Clontech), which was digested with *Xho*I and *BamH*I. The sequences of amino acid sites (44, 46, 272, 274) mutated in mLST8 were cloned into a pLVX-T2A-mCherry-Puro vector to construct mLST8 mutants for restoration experiments. All primer sequences are listed in Table S1.

### Cell culture and viruses

The PK-15 and HEK293T cell lines were purchased from the Cell Bank of the Chinese Academy of Sciences (Shanghai, China). The L929 (CCL-1) line was purchased from ATCC (USA). The cells were cultured in Dulbecco’s modified Eagle’s medium (DMEM) supplemented with 10% fetal bovine serum (FBS), 100 U/mL penicillin, and 100 µg/mL streptomycin and maintained at 37°C with 5% CO_2_. The following viruses were used: TGEV WH-1 strain (GenBank accession no. HQ462571.1), PDCoV strain CHN-HN-2014 (GenBank accession no. KT336560), and MHV A59 strain (GenBank accession no. MF618253.1).

### Transfection and infection

HEK293T cells were transfected with Jet PRIME (Poly Plus) reagent. The transfection process was performed according to the manufacturer’s instructions. For viral infections, WT and gene KO PK-15 cells were grown to approximately 80% confluence in a 12-well plate and incubated with TGEV at 37°C for 1 hour. After adsorption, the cells were washed with phosphate-buffered saline (PBS), which was replaced with fresh medium supplemented with 2% FBS, followed by incubation at 37°C in 5% CO_2_ for the indicated times. Infection was subsequently monitored by immunofluorescence and RT-qPCR.

### Construction of candidate gene-KO, stable gene rescue, and candidate gene overexpression cell lines

The sgRNA lentivirus corresponding to each candidate gene was transduced into PK-15-Cas9 cells. After 72 hours of transfection, cells with green fluorescent protein expression were identified by fluorescence-activated cell sorting. To generate rescue and overexpression cell lines, mLST8-KO and PK-15-Cas9 cells were transfected with lentivirus containing full-length mLST8 with site-specific mutations. After 72 hours of transfection, cells with mCherry expression were identified by fluorescence-activated cell sorting. Expression of the add-back mLST8 gene was confirmed by western blotting.

### Western blotting and antibodies

Cells were lysed in cell lysis buffer containing 1× protease inhibitor (Beyotime) for 30 minutes at 4°C, and cellular debris was removed via centrifugation (12,000× *g* for 15 minutes at 4°C). The lysed cells were denatured with SDS by heating at 95°C for 10 minutes, followed by incubation on ice for 5 minutes. After SDS-PAGE, the proteins were transferred onto a polyvinylidene fluoride (PVDF) membrane. The PVDF membranes were blocked for at least 3 hours using tris buffered saline plus tween-20 (TBST) with 5% milk. Subsequently, the PVDF membranes were incubated with primary antibodies in TBST overnight at 4°C or 3 hours at room temperature. Primary antibodies against the following proteins were obtained from Cell Signaling Technologies and used for western blot analysis: mLST8 (no. 3274, 1:1,000), 4E-BP1 (no. 9644, 1:1,000), S6K (no. 9202, 1:1,000), p-4E-BP1 (T37/46) (no. 2855, 1:1,000), p-4E-BP1 (S65) (no. 13443, 1:1,000), p-S6K (T389) (no. 9234, 1:1,000), p-ULK1 (S757) (no. 14202, 1:1,000), SQSTM1/P62 (no. 23214, 1:1,000), mTOR (no. 2983, 1:1,000), p-mTOR (S2448) (no. 5536, 1:1,000), ATF4 (no. 11815), and lipn1 (no. 14906). GAPDH (Beyotime, no. AF5009 1:5,000), β-actin (Beyotime, no. AF0003, 1:5,000), anti-mouse (Abbkine, no. A21010) and anti-rabbit (Abbkine, no. A21020), and secondary antibodies were applied at 1:5,000 and 1:10,000 dilutions, respectively, in TBST for 1 hour at room temperature. Proteins were detected with ECL Prime western blot detection reagents (GE Healthcare, United Kingdom).

### RT-qPCR

Total RNA was extracted from cells with TRIzol Reagent (Invitrogen). The isolated RNA was used to synthesize cDNAs with a PrimeScript RT Reagent Kit with gDNA Eraser (TaKaRa) in a total volume of 20 μL. Target genes were amplified in the following reaction systems: SYBR Green Mix at 5 μL, primers specific for the gene at 0.5 μL, and double-distilled H_2_O at 3 μL for a final reaction volume of 10 μL. PCR was performed for one cycle of 15 minutes at 95°C, followed by 39 cycles of 10 seconds at 95°C and 30 seconds at 60°C. The primer sequences used for RT-qPCR are shown in Table S1.

### Viral titers

Candidate gene-KO cells and WT cells were inoculated with TGEV in 24-well plates in triplicate. Cells were harvested at the indicated time points, and viral titers were determined on the basis of the median tissue culture infectious dose (TCID_50_).

### Immunofluorescence assay

Candidate gene-KO cells and control cells with susceptibility to viral infection were identified with an immunofluorescence assay targeting the TGEV N protein. Cells were infected with TGEV at different MOI values. Then, the cells were washed with PBS at 24 hpi. Subsequently, the cells were fixed with 4% paraformaldehyde for 30 minutes at room temperature, permeabilized with 0.3% Triton X-100 for 10 minutes, and then washed three times with 1× PBS. The permeabilized cells were blocked for 30 minutes with PBS containing 5% bovine serum albumin. The cells were incubated with TGEV N antibody (a rabbit anti-TGEV N protein polyclonal antibody was prepared in our laboratory) or anti-dsRNA antibody (SCICONS, no. 10010200, 1:1,000) at 37°C for 2 hours. Before incubation with the secondary antibody, the cells were washed three times with PBS for 10 minutes each. The secondary antibodies were Alexa Fluor 594 goat anti-mouse IgG (H + L) (Invitrogen, no. A-11005, 1:1,000) and Alexa Fluor 594 anti-rabbit IgG (H + L) (Invitrogen, no. A-11012, 1:1,000), and the cells were incubated at 37°C for 1 hour. Finally, the samples were washed three times with PBS, and the cell nuclei were counterstained with 4’,6-diamidino-2-phenylindole (DAPI) (Sigma, no. D9542) at room temperature for 2 minutes. Incubation with secondary antibodies and DAPI was carried out in the dark. Cell samples were observed and imaged with a fluorescence microscope (Thermo Fisher Scientific EVOS FL Auto).

### MTS assay, CCK-8 assay, and EdU fluorescence assays

To assess cell viability and cell proliferation, MTS, CCK-8, and EdU fluorescence assays were performed. The MTS assay was performed using the MTS assay kit (Abcam, no. ab197010). Cells were seeded into 96-well plates and incubated in a 37°C incubator for 12 hours. The culture medium was replaced with a fresh medium that contained the compounds at different concentrations and incubated continuously for 48 hours. Afterward, 10 µL of MTS reagent was directly added to each well, followed by incubation at 37°C in the dark for 2 hours. The absorbance values of the cells at 490 nm were measured to determine cell viability. The CCK-8 assay was performed using a CCK-8 kit (Beyotime, no. C0038). The cells were spread on 96-well plates and incubated for 24 hours. Each well was supplemented with CCK-8 solution, and the absorbance values of the cells were measured at 450 nm.

For the EdU fluorescence assay, KO and WT cells were seeded into 12-well plates. After 24 hours of incubation, the EdU cell proliferation assay was performed using the BeyoClick EdU cell proliferation kit (Beyotime, no. C0075S) with Alexa Fluor 555 according to the manufacturer’s instructions. Treated cells were visualized under fluorescence photomicroscopy. The proportion of EdU-positive cells was calculated using ImageJ software (three independent wells were imaged, and a random field of view per well was captured).

### TEM assay

mLST8 KO and PK-15 cells were infected with TGEV at an MOI of 1 for 12 hours. The cells were washed three times with precooled PBS, after which 3 mL of 2.5% glutaraldehyde (Servicebio) was added, and the cells were fixed for 2 hours at room temperature. After fixation, the cells were transferred to a 2-mL centrifuge tube. TEM samples were performed by Servicebio Company, and images were taken using an HZAU TEM platform (HITACHI, no. H-7650). Statistical analysis was performed on the reference DMV count (61−63), 40 cells were randomly observed, and the number of DMVs in each cell was counted. A two-tailed unpaired Student’s *t*-test was performed on the data.

### Drug treatment assay

JR-AB2-011 (MCE HY-122022), everolimus (MCE RAD001), and rapamycin (MCE HY-10219) were dissolved in dimethyl sulfoxide at a stock concentration of 10 mM and then frozen in aliquots at −80°C. The cells were incubated with the specified concentration of inhibitor for 2 hours and then infected with TGEV at an MOI of 0.01. Then, the cells were prepared for immunofluorescence assays at 24 hpi, and the supernatants were collected for viral titer assays.

### Virion attachment and internalization assay

WT and mLST8-KO cells were infected with TGEV (MOI = 5) and incubated at 4°C for 1 hour. For the virus attachment assay, the cells were washed three times with cold PBS (at 4°C) to remove unbound viral particles and harvested to extract viral RNA. The amount of viral RNA was determined by RT-PCR. For the internalization assay, the infected cells described above were further cultured with prewarmed DMEM for 1 hour at 37°C for another 1 hour. Subsequently, the cells were treated with 1 mg/mL pronase in cold PBS to remove the attached but uninternalized viral particles. After washing three times, the cells were lysed with TRIzol reagent to extract total cellular RNA, and the viral RNA was quantified by RT-PCR.

### Cholesterol detection assay

Total cellular cholesterol was quantified using a cholesterol assay kit (Solarbio, no. BC1985). PK-15 cells and mLST8 KO cells in six-well plates were collected and washed three times with PBS. An extraction solution was added at a ratio of cell number (10^4^): volume of extraction solution (mL) of 500–1,000:1. Subsequently, the cells were crushed by ultrasound in an ice bath for 3 minutes and centrifuged at 10,000 × *g* and 4°C for 10 minutes. Then, 180 µL of the centrifuged sample was transferred into a 96-well plate, 20 µL of working solution was added, and the plate was shaken well and incubated for 30 minutes at 37°C while protected from light. Then the samples were measured in a SpectraMax 190 spectrophotometer (Molecular Devices, California, USA) with the wavelength adjusted to 500 nm.

### LD staining

LD staining was performed using a lipid fluorescence staining kit (Nile Red Method) (Solarbio, no. G1264). PK-15 cells and mLST8 KO cells were plated on coverslips. The cells were treated with lipid fixative for 10–15 minutes at room temperature, followed by washing with PBS. Then, the appropriate amount of working stain solution was added to the cells, which were incubated at room temperature while protected from light for 10 minutes. Finally, the cell surfaces were washed with PBS while shaking for 1 minute. The coverslips were imaged with a confocal microscope (Zeiss, Germany). The LDs were quantified by ImageJ software analysis of different random fields of view.

### Statistical analysis

Statistical significance values were assessed using GraphPad Prism 8.0. Two-tailed unpaired *t*-tests were used to compare two unpaired groups. Multiple groups were compared by two-way analysis of variance. Unless otherwise stated, the data represent the mean ± standard deviation of experiments performed, at least, in triplicate.

## SUPPLEMENTAL MATERIAL

Supplemental File mbio.00899-23-s0001.docx Supplemental legends and figures.

Table S1 mbio.00899-23-s0002.xlsx Primers.

ASM does not own the copyrights to Supplemental Material that may be linked to, or accessed through, an article. The authors have granted ASM a non-exclusive, world-wide license to publish the Supplemental Material files. Please contact the corresponding author directly for reuse.
