# Supplementary material for: mLST8 is essential for coronavirus replication and regulates its replication through the mTORC1 pathway
Source: mBio. 2023 Jun 28;14(4):e00899-23. doi: 10.1128/mbio.00899-23 (PMC10470783; doi:10.1128/mbio.00899-23)
Supplement: Supplemental File — Supplemental legends and figures. [file mbio.00899-23-s0001.docx]

**
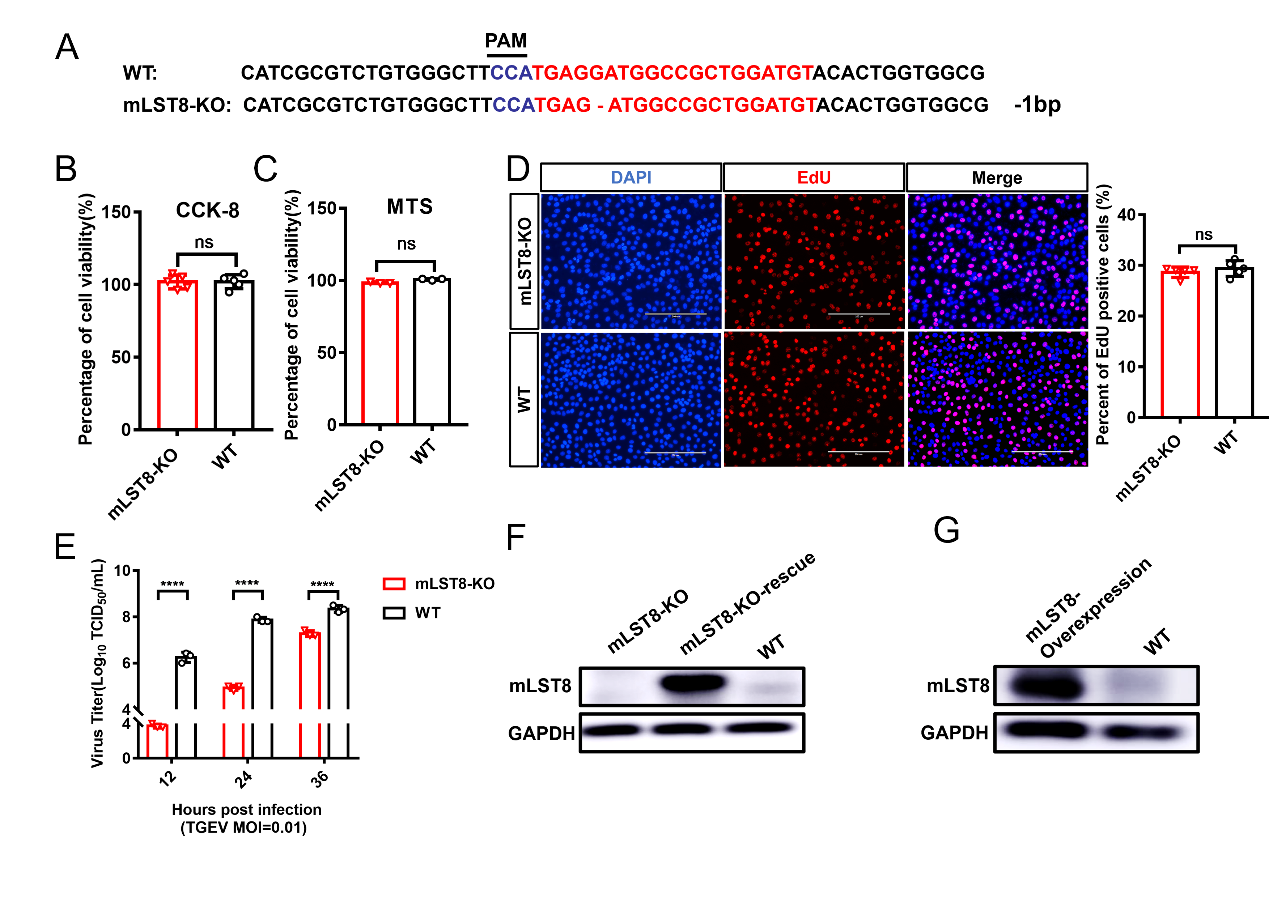
FIG S1 mLST8 is a host factor required for TGEV replication. (A)** Alignment of the nucleic acid sequences of mLST8 KO cells with those of WT cells. sgRNA-targeted sites are highlighted in red. The red characters “-” indicate the deleted bases. The PAM site is indicated by blue letters. **(B)** mLST8 KO and WT PK-15 cells were seeded into 96-well plates, and cell proliferation and viability were validated by CCK-8 assay at 24 hpi. **(C)** mLST8 KO and WT PK-15 cells were seeded into 96-well plates, and cell proliferation and viability were validated by MTS assay at 24 hpi. **(D)** There was no significant difference in the proportion of EdU-positive cells between the mLST8 KO cell line and WT cells. Left: representative pictures. Right: quantification of EdU-positive cells. **(E)** Multiple step viral growth assay. At different time points (12 h, 24 h, 36 h), the TGEV titers in mLST8 KO and WT cells infected with TGEV at an MOI of 0.01 were tested. **(F)** mLST8 KO, mLST8-KO-rescue and WT cells were cultured in 6-well plates, and the expression of mLST8 was detected by western blotting with anti-mLST8 antibody. **(G)** mLST8-overexpressing cells and WT cells were cultured in 6-well plates, and the expression of mLST8 was detected by western blotting with anti-mLST8 antibody. The means and SDs of the results from three independent experiments are shown. ns, not significant; ***, *P <*0.001; ****, *P <*0.0001.


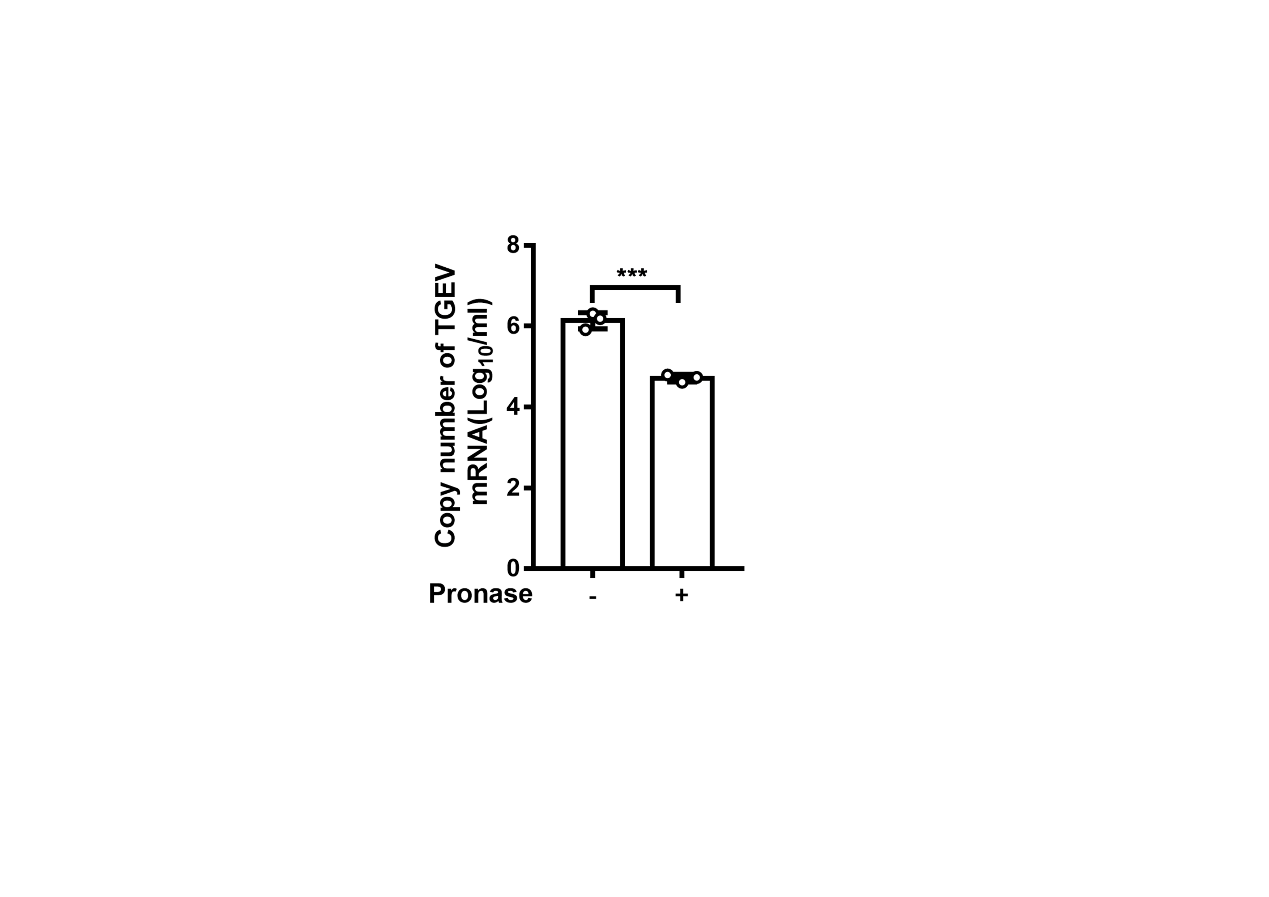


**FIG S2 Pronase removed attached particles that had not been internalized. (A)** PK-15 cells were infected with TGEV (MOI = 5) at 4°C for 1 h. The PK-15 cells were subsequently treated with PBS or PBS containing a final concentration of 1 mg/ml pronase. The cells were lysed with TRIzol reagent to extract total cellular RNA, and the viral RNA was quantified by RT–PCR. The means and SD of the results from three independent experiments are shown. ***, *P <*0.001.

**
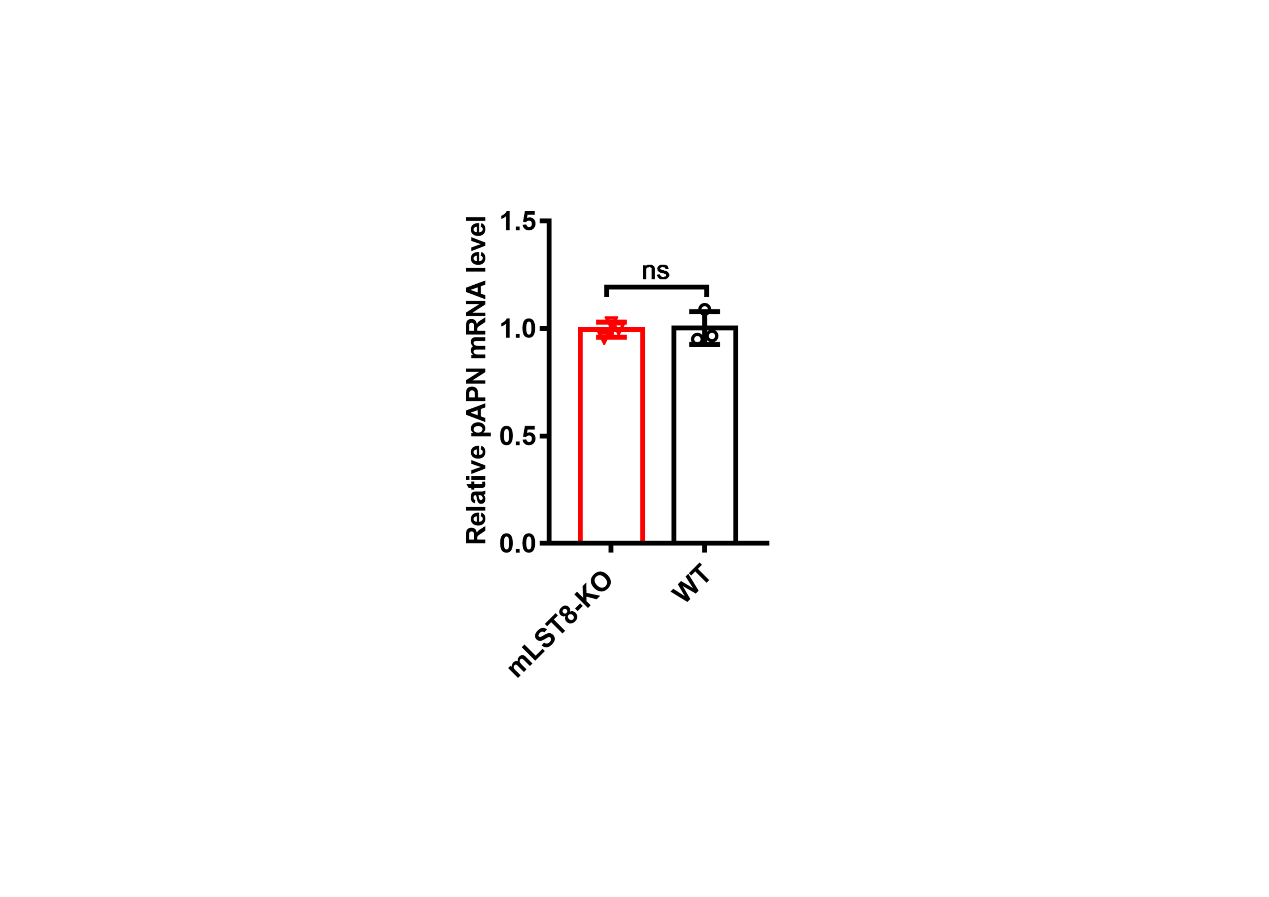
**

**FIG S3 Knockout of mLST8 had no effect on the expression of pAPN. (A)** The expression of pAPN in mLST8 KO cells and WT cells was detected by RT‒PCR. The means and SD of the results from three independent experiments are shown. ns, not significant.

**
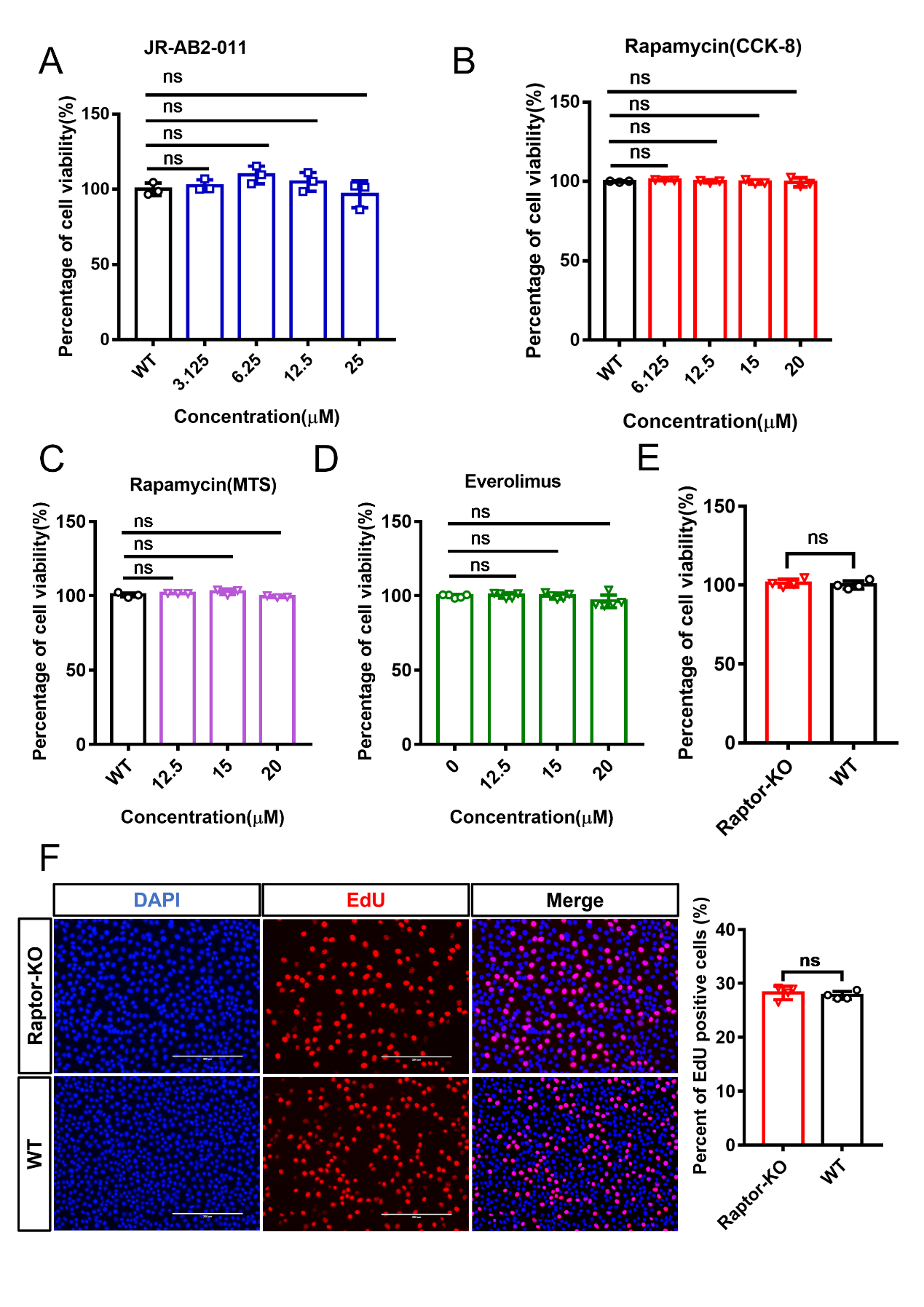
**

**FIG S4 Neither inhibitor treatment nor raptor KO affected cell viability or proliferation. (A)** PK-15 cells were treated with or without JR-AB2-011 for 48 h, and cell viability was verified by MTS assay. **(B and C)** PK-15 cells were treated with or without Rapamycin for 48 h, and cell viability and cell proliferation were verified by CCK-8 assay and MTS assay, respectively. **(D)** PK-15 cells were treated with or without Everolimus for 48 h, and cell viability and cell proliferation were verified by CCK-8 assay. **(E)** Raptor KO and WT PK-15 cells were seeded into 96-well plates, and cell proliferation and viability were validated by CCK-8 assay at 24 hpi. **(F)** There was no significant change in the proportion of EdU-positive cells between the Raptor KO cell line and WT cells. Left: representative pictures. Right: quantification of EdU-positive cells. The means and SD of the results from three independent experiments are shown. ns, not significant.

**
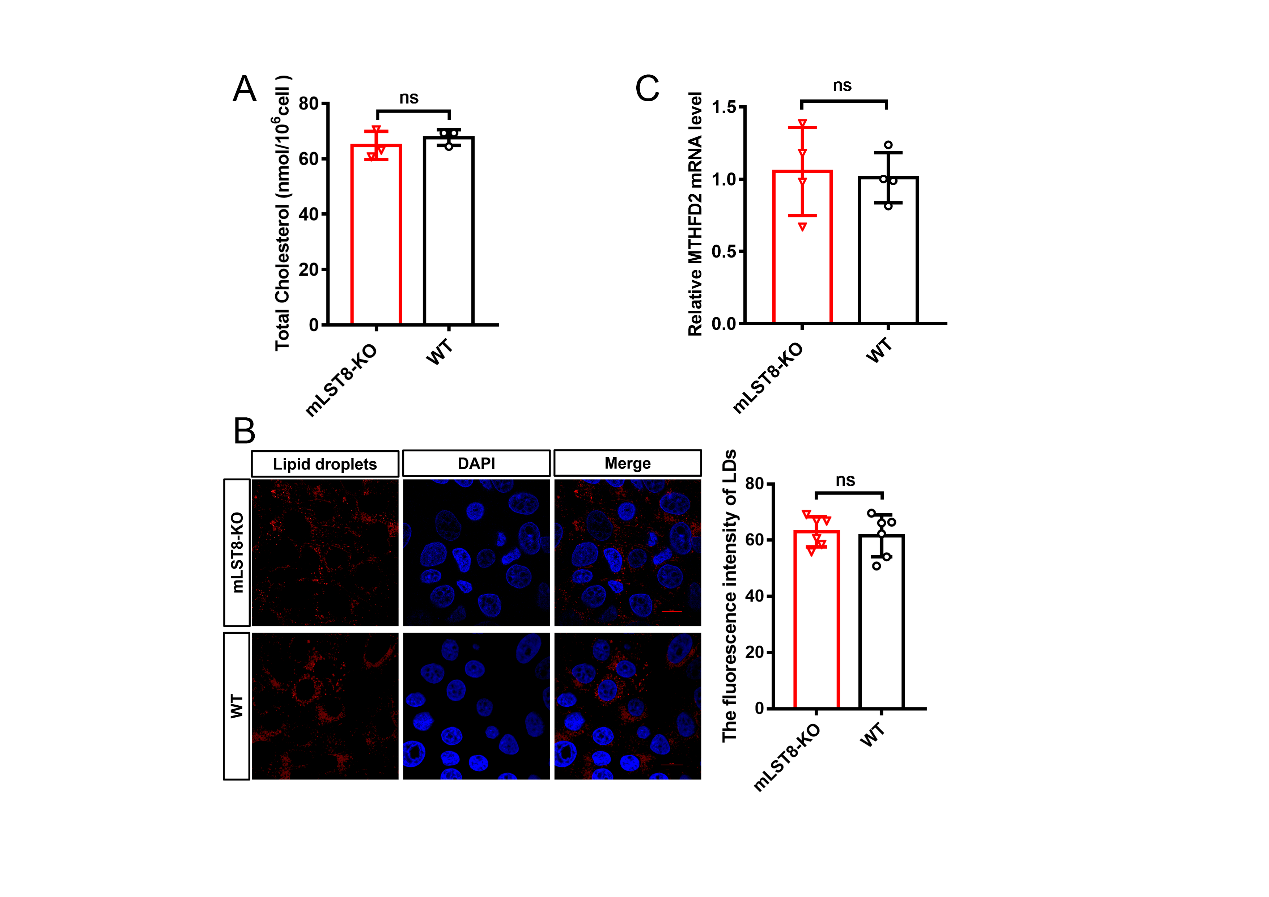
**

**FIG S5 The loss of mLST8 in PK-15 cells had no effect on lipid synthesis or MTHFD2 expression. (A)** Total cellular cholesterol in mLST8 KO and WT cells was detected with a cholesterol assay kit. **(B)** LDs were captured by fluorescence microscopy after [Nile Red](https://www.medchemexpress.cn/nile-red.html) staining (red), and nuclei were stained with DAPI (blue). **(C)** The expression of MTHFD2 in mLST8 KO and WT cells was detected by RT‒PCR. Data are shown as the means ± SDs from at least three independent experiments. ns, not significant.

**
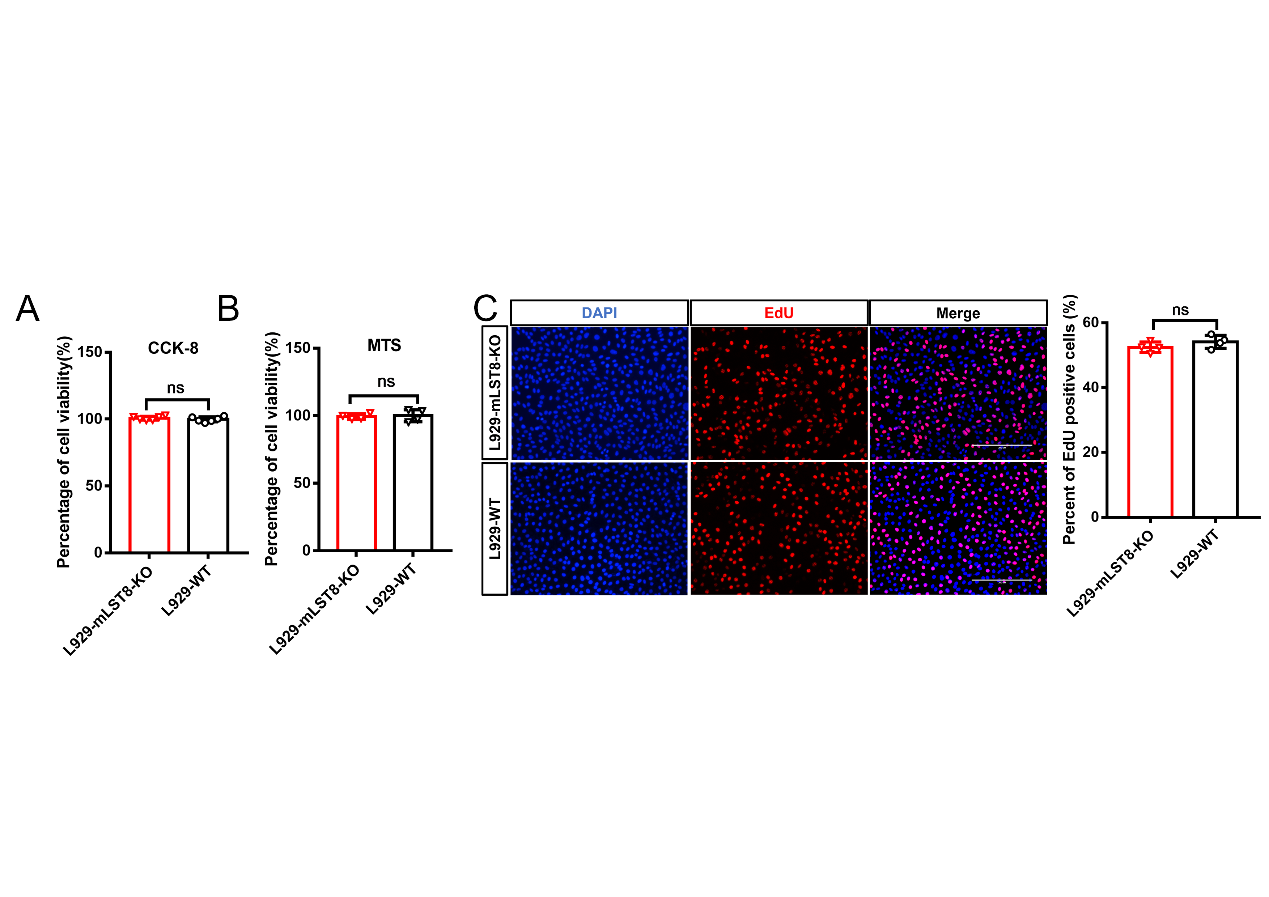
**

**FIG S6 The loss of mLST8 did not affect cell viability or cell proliferation in L929 cells.** **(A and B)** mLST8 KO-L929 and WT L929 cells were seeded into 96-well plates, and cell proliferation and viability were validated by CCK-8 assay and MTS assay, respectively, at 24 hpi. **(C)** There was no significant difference in the proportion of EdU-positive cells between mLST8 KO-L929 cell and WT L929 cells. Left: representative pictures. Right: quantification of EdU-positive cells. Data are shown as means ± SDs from at least three independent experiments. ns, not significant.
